# The impact of COVID-19 on autologous stem cell transplantation in multiple myeloma: A single-centre, qualitative evaluation study

**DOI:** 10.1007/s00520-022-07173-5

**Published:** 2022-06-03

**Authors:** Marquita Camilleri, Georgios Bekris, Govundeep Sidhu, Caroline Buck, Esma Elsden, Orla McCourt, Jackie Horder, Fiona Newrick, Catherine Lecat, Jonathan Sive, Xenofon Papanikolaou, Rakesh Popat, Lydia Lee, Ke Xu, Charalampia Kyriakou, Neil Rabin, Kwee Yong, Abigail Fisher

**Affiliations:** 1 University College Hospital Cancer Institute, Paul O’Gorman Building, 72 Huntley Street, London, WC1E 6DD UK; 2 Haematology Department, Addenbrooke’s Hospital, Cambridge University Hospitals NHS Foundation Trust, Cambridge, UK; 3 Department of Behavioural Science and Health, University College London, 1-19 Torrington Place, London, WC1E 7HB UK; 4 Therapies & Rehabilitation, University College London Hospitals NHS Foundation Trust, London, UK; 5 Haematology Department, University College London Hospital, University College London Hospitals NHS Foundation Trust, London, UK

**Keywords:** COVID-19, Autologous stem cell transplantation, Multiple myeloma

## Abstract

Autologous stem cell transplantation (ASCT) is standard of care in biologically fit, newly diagnosed multiple myeloma (MM) patients, offering better therapeutic outcomes and improved quality of life (QoL). However, with the UK’s 1st national lockdown on 23/03/2020, several guidelines recommended deferring ASCT due to risks of infection, with resource limitations forcing some units to suspend ASCT entirely. Such changes to patients’ treatment plans inevitably altered their lived experience during these uncertain times with expected impact on QoL. We conducted a qualitative study using semi-structured interviews to gain insight into MM patients’ understanding of their disease, initial therapy and ASCT, and their response to therapy changes. A clinical snapshot of how COVID-19 affected the MM ASCT service in a single UK institution is also provided, including changes to chemotherapy treatment plans, timing, and prioritisation of ASCT. Framework analysis identified 6 overarching themes: (1) beliefs about ASCT, (2) perceptions of information provided about MM and ASCT, (3) high levels of fear and anxiety due to COVID-19, (4) feelings about ASCT disruption or delay due to COVID-19, (5) perceptions of care, and (6) importance of social support. Example subthemes were beliefs that ASCT would provide a long-remission/best chance of normality including freedom from chemotherapy and associated side-effects, disappointment, and devastation at COVID-related treatment delays (despite high anxiety about infection) and exceptionally high levels of trust in the transplant team. Such insights will help us adjust our service and counselling approaches to be more in tune with patients’ priorities and expectations.

**Supplementary Information:**

The online version contains supplementary material available at 10.1007/s00520-022-07173-5.

## Introduction

Multiple myeloma (MM) is a haematological malignancy characterised by plasma cell accumulation in the bone marrow. Treatment is initiated after fulfilling the International Myeloma Working Group (IMWG) criteria for symptomatic disease [1] that follows a relapsing–remitting course requiring multiple lines of therapy. Although traditionally considered incurable, MM survival has improved substantially over the last three decades [2–5], predominantly due to improved anti-myeloma treatments and better supportive care allowing autologous stem cell transplantation (ASCT) in older patients [6]. Current guidance in the United Kingdom (UK) recommends high-dose melphalan (HDM) and ASCT in all newly diagnosed MM patients deemed suitable and biologically fit [7].

Nevertheless, HDM/ASCT remains non-curative and is not without risk [8]. Short-term complications include infections, nausea, anorexia, fatigue, and reduction in functionality and well-being for up to 12 months post-ASCT [9]. HDM/ASCT’s associated benefits and risks came into sharper focus during the COVID-19 pandemic as haematology patients were particularly vulnerable to infection [10] and advised to shield. Alternative management plans and new ways of delivering care were implemented wherever possible to reduce individual patient susceptibility [11]. Several clinical guidelines recommended deferring ASCT for MM [12–14] due to risks of infection further compounded by resource limitations from staff redeployment, shortage of ventilators and intensive care beds [15], and unavailability of blood products [16].

Aside from notable changes to healthcare delivery, the COVID-19 pandemic also profoundly affected our social order. Such enforced changes to patients’ treatment plans and requirement to shield were inevitably anticipated to alter their lived experience during these uncertain times, with expected impact on overall wellbeing. These new circumstances presented a unique opportunity to explore patients’ attitudes, perceptions, and expectations around MM and their treatment, including ASCT. Questionnaire data is rapid and economical to collect and is one of the best ways to achieve a wide population coverage. However, questionnaires can elicit cognitions about novel situations [17, 18] and pose limitations on the depth of information that can be gathered — able to explain “the what” but not “the why”. Qualitative studies are better poised to capture and understand how people make meaning and sense of health and illness [19, 20], while better providing in-depth information on how to best adjust to a post-pandemic environment [21].

Therefore, the aims of this study were to:

1. Provide a snapshot of how COVID-19 affected the MM ASCT service in a single-centre UK institution, including changes to chemotherapy treatment plans, timing, and prioritisation of HDM/ASCT
2. Gain insight into MM patients’ understanding of their disease, initial therapy and ASCT, response to therapy changes and their perception of COVID-19 infection risk.

It was hoped that, in addition to understanding how the COVID-19 pandemic altered MM patients’ perceptions of their health, the insight gained from this work would help us deliver an ASCT service more attuned to patients’ needs, expectations, and priorities.

## Methods

**Fig. 1:**
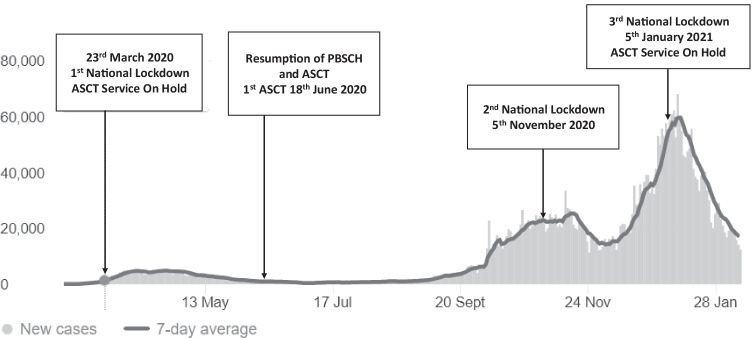
How the ASCT service at University College London Hospitals was affected by daily COVID-19 cases across the UK and consequent national lockdowns during periods of peak infection in the community

Clinical data was retrospectively collected from 115 newly diagnosed MM patients in a single centre in London, UK. All had a peripheral blood stem cell harvest for ASCT from December 2019 to January 2021. During this time, three national UK lockdowns led to the ASCT service being suspended twice (Fig. 1). Throughout this period, patients were discussed in multidisciplinary meetings with decisions made to delay or defer ASCT based on patient fitness, depth of response, and disease risk. High-risk MM is defined based on cytogenetic factors — either by the presence of an adverse-risk translocation [t(4;14), t(14;16), t(14;20)], and/or del(17)p. Patients with isolated chromosome 1 abnormalities were analysed separately.

All 115 patients consented to data registration with the European Society for Bone Marrow and Transplant (EBMT) to be used for the purposes of research, benchmarking, development of new and improved transplant procedures, and to improve the quality of these procedures. Being a service evaluation, additional ethical approval was not required as per Health Research Authority (HRA) guidelines [22]. However, patients who agreed to be interviewed consented to their contact details being stored in UCL’s Data Safe Haven and accessed by the research team to arrange and conduct interviews.

Thirty-nine patients from this cohort underwent human-based random selection and were contacted by the Myeloma Transplant team to determine interest in participating in a qualitative interview. Participants were sent an information sheet then given 5–7 days to decide whether to proceed with interview. The final sample size was based on data saturation guidelines for qualitative studies, whereby sample size is adequate when new information adds little or no change to the initial framework [23]. One-to-one telephone interviews were conducted by four health psychology researchers with training and experience in qualitative interviewing. Interviews were based on a semi-structured topic guide (Supplementary Material) designed to understand patients’ beliefs about ASCT, COVID-19-related treatment delays and levels of satisfaction with the information provided about these. Interviewers had no previous connection to patients and were independent of the clinical team and service. Interviews were audio-recorded, anonymised, and transcribed *verbatim* by a transcription service with an existing University College London (UCL) data sharing agreement.

## Analysis

Clinical data was presented using descriptive statistics generated by Microsoft Excel. Transcripts were checked for accuracy and framework analysis was carried out using Excel and nVivo software [24]. Framework analysis is suitable for a combined deductive and inductive approach, since codes can be used based on previous findings and opportunities for additional themes can be generated through the coding procedure [24].

A coding framework was first deductively created based on the interview schedule. Three researchers independently coded the same three transcripts to inductively modify the initial framework, and codes were adjusted following preliminary discussions. Further modifications were made following feedback and comments from the wider research group. The rest of the interviews were coded using the finalised framework and themes and subthemes were defined before writing the final report. The Consolidated Criteria for Reporting Qualitative Research (COREQ) checklist was the basis for the reporting of the qualitative part of this research [25].

## Results: clinical snapshot

**Fig. 2:**
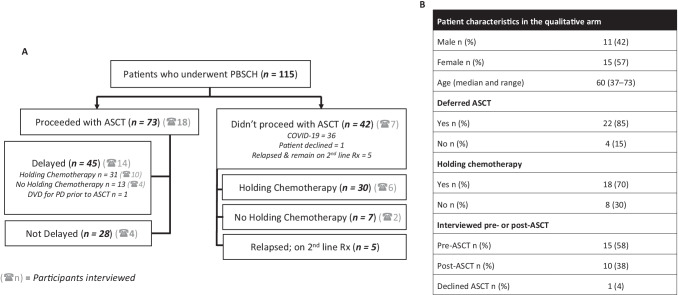
**A** A consort diagram illustrating how the COVID-19 outbreak influenced newly diagnosed MM patients’ myeloma treatment pathway. **B** Summary of patient characteristics in the qualitative study. MM, multiple myeloma; ASCT, autologous stem cell transplantation; Rx, treatment; DVD, daratumumab-bortezomib (Velcade)-dexamethasone

**Fig. 3:**
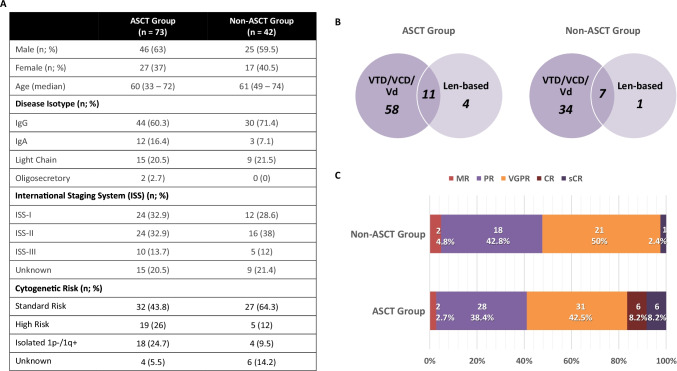
Summary of the clinical results including summary of patient characteristics (**A**), induction treatment regimens (**B**), and response to induction chemotherapy (**C**)

Of the 115 patients due to proceed to ASCT during the pandemic, 73 (63%) proceeded to ASCT and 42 (37%) were deferred indefinitely (ASCT^def^) (Fig. 2A). Disease and demographic characteristics were similar between the two groups (Fig. 3A). However, patients in the ASCT group were more likely to have cytogenetic features associated with a worse prognosis (Fig. 3A).

Both groups were predominantly treated with bortezomib-based induction, though 15% of ASCT and 17% ASCT^def^ patients were switched to a solely oral (lenalidomide-based) treatment regimen in line with national guidance [26] to reduce hospital attendances (Fig. 3B). The duration of induction therapy was median 6 cycles in both groups. While most patients had a good response to induction treatment, salvage chemotherapy was needed in 10/73 (13.7%) ASCT and 3/42 (7.1%) ASCT^def^ patients due to induction-resistant MM requiring urgent disease control before stem cell harvest (Fig. 2A). 28/73 (38.4%) in the ASCT group had no delay to ASCT having undergone transplantation within a median time of 8 months (range 5–13) from start of induction. None of these patients were put on holding chemotherapy. 45/73 (61.6%) had a delayed upfront ASCT within a median of 11 months (range 5–17) from start of induction, of whom 31 were put on holding chemotherapy while the other 13 remained treatment-free (Fig. 2A). In the 42 ASCT^def^ patients, 5 relapsed within less than 6 weeks of stem cell harvest and received 2nd line therapy, 30 were put on holding chemotherapy as per NHS England COVID-19 interim guidance [26], and 7 remained treatment-free post-induction (Fig. 2A).

## Results: qualitative interviews

Of 39 patients approached, 26 (67%) took part in interviews. Figure 2A highlights where these patients were in their treatment pathway, with their characteristics presented in Fig. 2B. Participants were aged 37–73, with a mean age of 61 years (SD = 7). There were 42% males and 58% females.

### Themes and subthemes

Six themes and 12 subthemes were identified from the data; these are presented in Table 1 with supporting quotes. Overarching themes were (1) beliefs about ASCT, (2) perceptions of information provided about MM and ASCT, (3) high levels of fear and anxiety due to COVID-19, (4) feelings about ASCT disruption or delay due to COVID-19, (5) perceptions of care, and (6) importance of social support.

1. **Beliefs about ASCT**

1.1. ***Long remission***

Almost all patients viewed the prospect of ASCT as overwhelmingly positive, with long remission periods being a very commonly perceived benefit. Most had a specific estimated remission time in mind, and some estimates were long (e.g. 20 years). Patients often mentioned their doctor as a reason for their beliefs about remission. Where the doctor was the main source, estimates of remission time tended to be shorter. However, when participants described very long remissions, this often had a “word of mouth” or almost mythical quality to it; someone they had “heard of once”. Examples of people seen in the media who had long remissions were also commonly cited.

1.2. ***Break from chemotherapy and side-effects***

A very common perceived benefit of ASCT was having a period without chemotherapy and all of its associated negative side-effects. Participants highly valued and welcomed the potential of a treatment break.

1.3. ***Return to normality***

Linked to their diagnosis and treatment impacting identity and quality of life, participants perceived that ASCT would allow them to return to who they had been before myeloma and its treatments, allowing them a substantially better quality of life. Many used terms relating to returning to their pre-myeloma “normality”.

1.4. ***Tough treatment and infection risk***

When probed about negative aspects of ASCT, one participant talked about time away from family being hardest. However, participants generally described tough/grueling treatments and increased likelihood of infections, while acknowledging the higher risk due to COVID-19. This period of feeling weak and unwell was expected to be temporary, and a number of participants said they did not perceive any negative aspect. Even when participants understood and considered the additional risks of COVID-19, they commonly reflected that the benefits of ASCT still outweighed the additional risks posed by COVID-19.

2. **High levels of fear and anxiety due to COVID-19
**

2.1. ***Awareness of elevated vulnerability***

Patients had a clear understanding that MM and its treatments made them particularly vulnerable to severe illness if they caught COVID-19 (many described feeling sure they would die if they caught it) and as a result were constantly anxious. They described that the precautions they had to take increased their levels of anxiety to some extent as the thought of doing something wrong and catching COVID-19 escalated their fears.

2.2. ***Sense of unfairness — cancer then COVID-19***

Participants described a strong sense of unfairness of having to cope with COVID-19 after already having to deal with their myeloma diagnosis. They described how they had been left with fear and uncertainty around their myeloma treatment and progression, and now were having to deal with the additional burden and uncertainty of a pandemic.

2.3. ***Major impact of shielding: isolation versus safety***

Many patients were profoundly affected by shielding. They described missing social contact and how the process negatively impacted their mental health. This was particularly pronounced in people who lived alone*.* However, some also described an element of reassurance and safety by shielding, and that this was a way of mitigating the fear and anxiety of catching COVID-19. Participants discussed guilt and anxiety around the impact that their need to shield had on their family members. However, many also discussed anxiety caused by their family members being unable to shield and having to leave home and interact with others to work.

3. **Feelings about ASCT disruption or delay due to COVID-19
**

3.1. ***Disappointment and devastation***

Participants generally expressed high levels of disappointment and distress around delays to their ASCT. They described having prepared themselves both psychologically and practically to have treatment. This being taken away was extremely hard to deal with. However, one reported feeling relieved, because they had felt uncomfortable about going into hospital during the pandemic.

3.2. ***Desire to regain control overriding the fear of COVID-19***

Participants described that not knowing when transplants might resume (on top of the uncertainty around the pandemic) led to a feeling of total loss of control, and high levels of uncertainty that contributed to their anxiety. The need to feel in control, coupled with positive beliefs around ASCT, in some cases, led to patients evaluating their personal risk of COVID-19 and wishing to go ahead with ASCT regardless.

4. **Experience of information provision**
  Participants described their experiences of the consultations with the clinical teams where information about their treatment changes/delays were provided. The majority did not feel fully informed, but very rarely blamed the clinical team for this. Some described how they felt shocked so could not take the information in. Many said they chose not to be fully informed because the information was hard to deal with; “ignorance is bliss”. Occasionally, patients reported not feeling informed due to structural barriers like language. Only one felt they had all the information they needed.

5. **Perceptions of care**

5.1. ***High levels of trust in the transplant team and appreciation for the NHS***

Nearly all patients expressed extremely high levels of trust in the transplant team and felt this strongly despite delays. Most patients expressed their appreciation and gratitude for the NHS. They stated that the health professionals were supportive, and they felt grateful for the level of care they had received despite disruptive treatment changes. However, there were comments about administrative issues like lack of coordination of appointments that they attributed to pandemic-related changes, but that caused frustration.

5.2. ***Remote appointments well accepted***

On the whole, participants reported a positive experience of conducting general consultations remotely as this meant they did not have to travel and expose themselves to unnecessary risk. Participants who had a face-to-face consultation raised the issue of new barriers to communication created by COVID-19, such as masks, affecting doctor-patient communication.

6. **Importance of social support**

A common thread throughout the interviews and interwoven with multiple themes was the importance of social support, from identifying coping mechanisms to making shielding tolerable. Participants talked about how speaking to others who had been diagnosed with myeloma was extremely helpful. They also described the importance of family and friends in “getting them through” and giving them hope.

**Table 1:** Main themes and subthemes derived from the framework analysis

Themes	Subthemes	Example quotes
1. Beliefs about ASCT	1.1 Long remission	*The benefit (of ASCT) is…longevity basically. But as the doc said, it’s not something you die of, but it’s something you die with, so, that’s fine, but if I die with it, I hope it’s not for another 15 or 20 years*
	1.2 Break from treatment and side-effects	*it gives you, from my understanding, it gives me three to five years.. without having to have more treatment, so there is implicit benefit in that*
	1.3 Return to normality	*I have to do everything because I have no life quality. I have no life quality; the transplant will make my life different* *I see it as a positive (ASCT). I’ve had a very tough year so all I’m after really is having a kind of, is having some sort of normalisation*
	1.4 Tough treatment and infection risk	*The disadvantage of transplant, I suppose all there is just the very small risk that one dies of side-effects, like infections. But I can’t really see any disadvantages, other than the slight risk to mortality*
2. High levels of fear and anxiety due to COVID-19	2.1 Awareness of elevated vulnerability	*very, very difficult because I was absolutely petrified that I would get Covid and I was actually convinced that I would probably die. I am in a very vulnerable position. It is always on your mind*
	2.2 Sense of unfairness – cancer then COVID	*that was another worry, as if you didn’t have enough to cope with the cancer, you then had to think…..anyone who came to the door you’d think, well I don’t want to touch that in case I catch Covid..yeah the anxiety was off the roof* *I am scared (of COVID). I just hope that as each day goes by I will stand a chance. I’ve been through so much since the diagnosis that I just want a period of my life back and not to feel like I have felt for the last year*
	2.3 Major impact of shielding: isolation versus safety	*I think psychologically that was probably the part, my lowest part, because I never, I couldn’t leave the house, nobody could visit me for four months, I was completely shielded and I think psychologically, I was just scared* *When I’m staying inside, I’m happy, I’m okay, but outside I am very much uncomfortable*
3. Feelings about ASCT disruption or delay due to COVID-19	3.1 Disappointment and devastation	*I was devastated because I was psyched up ready to go. I was terribly, I am not going to deny it, I was very, very anxious* *I’ve had a lot of disappointments in my life but that is like someone cutting your arm off*
	3.2 Desire to regain control overriding the fear of COVID	*It was the uncertainty of when the transplant was going to happen because you have no control over anything really, but… my ability to plan, my ability to—we sent a rather pleading email that I understood exactly what my risks were and I didn’t want to delay the transplant* *[I was told] If you get Coronavirus, you will die, simple as that. I said, Look, we’ve already had a chat about this scenario, I’m well aware of that and we’re all prepared—I’m especially prepared to take the risk, rather than go on endless months of holding chemo*
4. Experience of information provision		*I never really asked a lot of questions about, because it was such a shock…I didn’t want too much detail and.. there is a few more questions about this treatment that I probably have to ask, because slowly, slowly you build up your confidence and your resilience but it’s quite hard at the beginning* *Some of the things are very clear but even with respect sometimes I think the less I know the better it is, ignorance can be bliss in this instance because otherwise you lose your mind*
5. Perceptions of care	5.1 High levels of trust in transplant team and gratitude for the NHS	*I just, I trust them. I just trust them immensely, 100%* *I’d like to thank the staff: nurses; doctors; the cleaners; the everybody and between for their hard work and dedication that they put in to help people like me. From the bottom of my heart* *I would prefer to have the phone consultations than to take the risk of going in to the hospital environment, so I’m happy with it*
	5.3 Remote consultations well received	*The barrier (in face to face appointments) at the moment, is wearing masks; it’s difficult to have a thoughtful conversation with somebody when your glasses are steamed up and their glasses are steamed up and you’ve got masks on. I think the way they changed and moved to the telephone consultation, is much better* *It’s not the same, having a telephone consultation as having face-to-face consultations – totally different. But it worked for me*
6. Importance of social support		*If I go and see my son and lovely daughter-in-law…and my grandson is the joy of my life really. He saved me actually because I didn’t realise how down I was, but he’s given me some kind of hope for the future, yeah*

*ASCT*, autologous stem cell transplantation; *MM*, multiple myeloma; *NHS*, National Health Service

## Discussion

In our initial clinical descriptive analysis, over 75% of transplant-eligible, newly diagnosed MM patients had their treatment significantly changed during the COVID-19 pandemic; 39% of patients had a delayed ASCT and 37% having no ASCT upfront. Of those whose ASCT got delayed/deferred, 75% were put on holding chemotherapy to mitigate the risk of MM relapse; this excludes the 6 patients who needed 2nd line chemotherapy for confirmed disease progression. Participants presented high levels of fear and anxiety around COVID-19, with patients having a clear understanding of their particular vulnerability to infection. However, this understanding conflicted with their feelings around some changes to their care implemented to ensure their safety. COVID-19-specific precautions and shielding made patients miss social contact which negatively affected their mental wellbeing. More strikingly, the heightened anxiety around COVID-19 was not enough to put some patients off having an ASCT during this high-risk period.

Possibly explaining this conflicting dichotomy is our participants’ beliefs that ASCT is a route to a long remission and increased longevity. They also valued the chemotherapy-free period that previously tended to follow an ASCT before lenalidomide maintenance became NICE-approved in March 2021 [27]. This time off treatment implied a return to normality back to a life before their MM diagnosis, and a break from treatment-related side effects which participants associated with a better QoL. With so much expectation hinging on the ASCT, COVID-19-related treatment adjustments caused much disappointment and devastation amongst all participants. Also, not knowing when the ASCT service was going to resume led to patients feeling a total loss of control and high feelings of uncertainty. For most patients, information provided by healthcare staff about MM and its treatment was difficult to process, either because of feelings of shock that came with their new diagnosis or personal preference. Other aspects of communication were more positive, with patients continuing to feel well-supported by healthcare providers even when services became more remote and telephone-based during the COVID-19 pandemic. Remote appointments came with the advantages of safety, convenience, and eliminating new barriers to face-to-face interactions caused by masks and visors. Nevertheless, patients were negatively affected by the additional strain COVID-19 placed on healthcare administrative logistics.

Our qualitative analysis expanded upon prior research reporting initial shock at diagnosis, with negative emotional problems complicating ambiguous perceptions that MM is both life menacing and manageable [28, 29]. Our findings also highlighted additional anxieties COVID-19 placed on patients already struggling with their cancer; this will require special attention when counselling newly diagnosed MM patients in a post-COVID-19 environment. In the absence of cure, the broad aims of MM therapy are to control disease and prolong survival while maximising QoL [30]. In MM patients, health-related QoL is predominantly influenced by therapy, with improvements in disease-related symptoms potentially being offset by drug toxicity [31]. This is particularly relevant in the immediate stages of ASCT, since MM patients would not commonly be exposed to traditional cytotoxic chemotherapy before the HDM preceding transplantation. Previously published literature demonstrated that ASCT leads to a short-term deterioration in health-related QoL and symptom burden in MM [32]. However, this improves to baseline health status within 1 to 2 months post-ASCT, with long-term QoL improving further thereafter [32]. This improvement has been attributed to superior disease control which patients hope will translate to an increased life expectancy [28, 33]. However, it may also reflect the benefits that come with the treatment-free period that used to follow ASCT. During this plateau phase, many patients enjoy reasonable QoL and wellbeing with several patients returning to pre-morbid lifestyles [30]. Our research reinforces this, with the perceived benefits of ASCT being so compelling that most patients were willing to take on the additional infection risk posed by COVID-19. This is despite fully appreciating their increased infection risk status. Yet, with lenalidomide maintenance post-ASCT now being standard of care, this highly desirable “chemotherapy-free period” will only last for 3 months after transplantation, during which QoL will be suboptimal while recovering from ASCT. As MM treatment strategies become more continuous both at diagnosis and at relapse, healthcare professionals should address these expectations when counselling patients about the benefits of ASCT.

Healthcare professionals providing inadequate clinical information to MM patients were previously reported [34] and also emerged in our study. The benefits of adequate information provision are well described [35]. They include reduced anxiety and fear about cancer and treatment as well as improved QoL, therapy adherence, psychological wellbeing, daily functioning, and better engagement in decision-making [35]. Nevertheless, unmet information needs remain a challenge in current cancer care with little evidence on how barriers in information exchange can be targeted [35]. Shared decision-making healthcare models promoting patient empowerment are advocated as the optimal communication model for cancer patients [36]. However, we observed clear psychological barriers to understanding complex clinical information, which may be better addressed with efficacious and timely professional psychotherapeutic support.

This study is one of the first to respond to the need of qualitative patient data during a pandemic [21], and the multidisciplinary nature of this analysis carried out by multiple researchers enhances the validity of our results and conclusions [37, 38]. One study limitation is that participants were recruited from a single centre which may not be representative of all UK MM transplant services [39], including geographical variations in COVID-19 prevalence and shielding guidance. While qualitative study designs provide in-depth data on patients’ experiences and perceptions, they can come with the caveat of social desirability and interviewer biases [38, 40]. Also, the lack of supportive quantitative data that is sometimes used in mixed-methods research limits our ability to provide a broader and more multidimensional picture of the problems investigated in this study [41, 42].

## Conclusion

The COVID-19 pandemic made us reflect on long-established MM treatment pathways, especially for transplant-eligible patients. New COVID-19 adapted guidelines, alongside new ways of perceiving and balancing ASCT risk, led to a substantial proportion of MM patients having a delayed or indefinitely deferred ASCT. Such enforced changes gave an opportunity to explore patients’ attitudes, perceptions, and expectations around MM and ASCT. Even with a well-known increased infection risk, patients viewed ASCT as an important component of their MM care. Any risks were outweighed by the perceived benefits of a long, “chemotherapy-free” remission, which is effectively expected to result in increased longevity and improved QoL. With MM treatment strategies becoming more continuous at both diagnosis and relapse, healthcare professionals should manage these specific expectations when counselling patients about the benefits of ASCT. Efforts towards more effective communication, better information delivery, and more streamline treatment pathways can be guided by insights gained in this study, allowing for adjustments to myeloma services to be more in tune with patients’ needs, priorities, and expectations.

## Supplementary Information

Below is the link to the electronic supplementary material.

Supplementary file1 (DOCX 15 KB)

## Data Availability

Can be made available upon request.
