# Supplementary material for: The impact of COVID-19 on autologous stem cell transplantation in multiple myeloma: A single-centre, qualitative evaluation study
Source: Support Care Cancer. 2022 Jun 3;30(9):7469–79. doi: 10.1007/s00520-022-07173-5 (PMC9163289; doi:10.1007/s00520-022-07173-5)
Supplement: Supplementary file 1 — Supplementary file1 (DOCX 15 KB) [file 520_2022_7173_MOESM1_ESM.docx]

**Supplementary Material**

**Appendix A - Interview Guide**

This guide helps the researcher/interviewer cover the key topics, concerning the aims of the study, during the interview. Nonetheless, the interview should be based in a respondent-sensitive nature, to allow the researcher to be more flexible through the questions and to give the space to the participant to raise additional issues.

Because of the chronic and severe character of MM, Interviewers must take into consideration the sensitive subject of the interview.

**Before-the-interview guidelines**

a) Ask permission to record the conversation.

b) Assure confidentiality

c) Ask participant to be as open and honest as they can be

**Introduction**

*“Hello, my name is [name] and I am [MSc student, researcher etc]. I am helping the Myeloma Team conduct these one-on-one interviews for their research project which is looking into how COVID-19 has and is still affecting myeloma transplant patients. We are independent from Myeloma team and because we are not part of your medical team, we do not know anything about your disease or your treatment, and we would not be able to answer any questions you may have about your myeloma or your treatment plan. However, I am sure the medical team will be more than happy to answer those questions should you happen to have any.”*

***Question 1***

What symptoms lead you to be diagnosed with myeloma?

Prompt: Tell me about your journey until diagnosis

***Question 2***

What was your experience with your initial treatment (chemotherapy +/- radiotherapy if given) before meeting the transplant team at the hospital?

***Question 3***

You were referred for consideration for transplantation by your hospital. What do you see as benefits of having a transplant for myeloma, and what do you see as the negative aspects?

Prompts: What makes you think that?

What is your source of information you are based on?

***Question 4***

Was the initial transplant chat with the Myeloma Transplant team at the hospital a face-to-face consultation or a phone consultation? (closed-ended question: a) face-to-face b) phone consultation)

How did you feel after having that consultation/the initial transplant chat?

Prompt: What was your experience of that process?

***Question 5***

What is your understanding of what you have been told by the Myeloma Transplant team then?

Prompt: Did you feel you got all the information you needed?

Did the information from the clinical team match what you have been told by others or what you might have read on patient information leaflets or the internet?

***Question 6***

How did your myeloma journey change during the COVID-19 pandemic?

Prompt: Were there any changes concerning your hospital appointments, your consultations or anything else you would like to share?

***Question 7***

How did this make you feel?

Prompts: How did you feel about the risk of COVID-19 infection?

What made you feel most anxious?

How was the shielding aspect for you?

How did that make you feel?

**Question 8**

How has COVID-19 affected your views about having transplant?

Prompt: Do you feel the risk of a transplant is greater now than before the COVID-19

pandemic?

**Question 9**

How has COVID-19 affected your treatment planned by your doctors?

Prompt: Is there any way the clinical team could have helped with this?

Do you have comments on any new treatments you have received and how do you

feel about this?

**Question 10**

How do you feel about any changes to your transplant pathway?

Prompts: What do these changes mean to you?

How do you feel about them now?

How did you feel about your treatment changes then (when they were announced to

you)?

**Final Question**

Is there anything else you would like to tell us that we haven’t discussed?

**Concluding guidelines**

a) Thank the participant for their time

b) Let them know where they can contact you in the future for any possible additional comments or clarifications
